# The use of diversity indices for local assessment of marine sediment quality

**DOI:** 10.1038/s41598-021-94636-0

**Published:** 2021-07-22

**Authors:** Shinya Hosokawa, Kyosuke Momota, Anthony A. Chariton, Ryoji Naito, Yoshiyuki Nakamura

**Affiliations:** 1 Marine Environmental Information Group, Port and Airport Research Institute, 3-1-1 Nagase, Yokosuka, Kanagawa, 239-0826 Japan; 2 Central Laboratory, Marine Ecology Research Institute, 300 Iwawada, Onjuku, Isumi, Chiba, 299-5105 Japan; 3 Department of Biological Sciences, Macquarie University, Macquarie Park, NSW 2113 Australia; 4 Coastal, Marine and Disaster Prevention Department, National Institute for Land and Infrastructure Management, 3-1-1 Nagase, Yokosuka, Kanagawa, 239-0826 Japan; 5 Department of Civil Engineering, Yokohama National University, 79-5 Tokiwadai, Hodogaya-ku, Yokohama, Kanagawa, 240-8501 Japan

**Keywords:** Biodiversity, Marine biology

## Abstract

Diversity indices are commonly used to measure changes in marine benthic communities. However, the reliability (and therefore suitability) of these indices for detecting environmental change is often unclear because of small sample size and the inappropriate choice of communities for analysis. This study explored uncertainties in taxonomic density and two indices of community structure in our target region, Japan, and in two local areas within this region, and explored potential solutions. Our analysis of the Japanese regional dataset showed a decrease in family density and a dominance of a few species as sediment conditions become degraded. Local case studies showed that species density is affected by sediment degradation at sites where multiple communities coexist. However, two indices of community structure could become insensitive because of masking by community variability, and small sample size sometimes caused misleading or inaccurate estimates of these indices. We conclude that species density is a sensitive indicator of change in marine benthic communities, and emphasise that indices of community structure should only be used when the community structure of the target community is distinguishable from other coexisting communities and there is sufficient sample size.

## Introduction

Diversity indices are used to measure the richness and evenness of species diversity^1^. Because these indices aid in the interpretation of changes in benthic communities, they can be used as ecological indicators of marine sediment quality^2^, and have advantages over several other sediment assessment approaches, such as chemical and toxicological evaluations, because of their more realistic application under field conditions^3^. For marine benthic invertebrate communities, diversity indices are usually estimated from counts of individuals obtained by using areal unit samplers such as quadrats or bottom samplers e.g. ^4,5^. Species richness, the Shannon index, and Pielou evenness are three diversity indices that are commonly estimated from these variables, and they all tend to decrease with increasing environmental contamination^6^. In addition, areal species richness (also known as species density), which normalizes a community’s species richness by the area sampled^7^, is also used to assess changes in benthic communities.

However, small sample size (i.e. not enough individuals sampled to estimate true diversity) can introduce considerable uncertainty. Although small sample size is not generally a problem when estimating species density because of the way the index is defined, it often causes uncertainty in other diversity indices. For example, species richness follows an increasing curve (called a rarefaction curve) as the number of reference individuals increases^8^. Indices for community structure, which become asymptotic at infinite sample size (i.e. for the true relative abundances^9^), have been shown to have rarefaction curves similar to those for species richness^9^. The exponential of the Shannon index and the inverse Simpson’s concentration index are representative of other indices that show rarefaction curves. Pielou evenness is also understood to be an index of community structure, because it is calculated from the Shannon index. These indices can substantially underestimate the true values when the number of individuals observed is small^10^.

Assessments of sediment quality may serve to test whether diversity indices respond to changes in sediment quality in a community. However, marine invertebrate communities are often also affected by small-scale gradients in other environmental factors^11,12^. If an appropriate community for testing cannot be identified, real community responses can be masked by variabilities in coexisting communities^2,6^. When the pattern of community variability is predictable, it may be relatively easy to isolate a target community and test its response to sediment contamination. However, because the spatial distribution of benthic communities is a complex mosaic in areas affected by marine urbanization^13^, it can be difficult to identify and distinguish between a target community and other communities.

From these perspectives of sample size and community variability, we wondered whether diversity indices could work as indicators of sediment quality under real field conditions. To address this, we proposed two questions. First, although species richness, the Shannon index, and Pielou evenness are known to decrease with sediment degradation^6^, we chose to investigate whether these responses reflect real changes in benthic communities, considering that marine benthic invertebrates are generally not abundant in degraded sediments. Second, in cases where decreases in these indices are real, we examined whether these indices are sensitive to changes in marine benthic invertebrates at the local scale that is most often the focus of assessment efforts.

**Figure 1:**
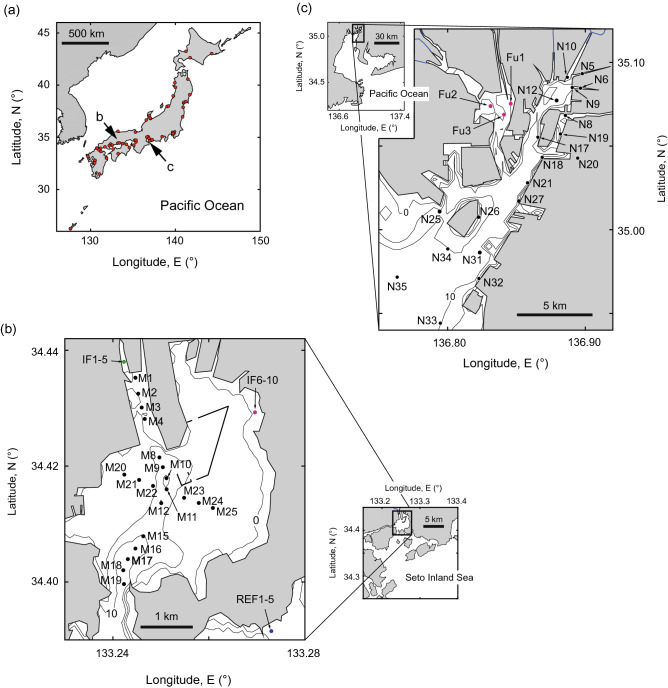
Maps of sampling locations. The number of sampling locations was (**a**) 65 across Japan, (**b**) 30 in Matsunaga Bay, and (**c**) 22 in Nagoya Port. IF1–5 (green) and IF6–10 (magenta) in (**b**) indicate the locations of intertidal flats at the mouth of a small river and in the inner bay, respectively. The location marked REF1–5 (blue) in (**b**) is a reference intertidal flat outside the bay, which was analysed in Supplementary Fig. S6. Locations Fu1–3 in (**c**) are sampling locations on the Fujimae intertidal flat. This map was created based on coordinate data from Google (http://www.gis-tool.com/mapview/maptocoordinates.html).

In this study, we chose to focus on three diversity indices: taxonomic density, the inverse Simpson’s concentration index, and Pielou evenness. Although the Simpson’s concentration index is better known than its inverse, we chose the inverse index (also known as “the effective number of species”) because of its ease of interpretation^14,15^. Hereafter, we will refer to the inverse Simpson’s concentration index simply as “Hill–Simpson diversity,” as was done in Roswell et al.^15^. We explored how to robustly assess sediment quality by estimating these three diversity indices with high confidence (i.e. with appropriate sample size and consideration of community variability) in our target region, Japan. Our investigation was performed at the regional scale (Fig. 1a) and in two local areas at intermediate latitudes within this region (Fig. 1b,c). The regional dataset was previously analysed such that species density can be related to a group of sediment variables, which include measurements of softness and contaminants^16^. We reanalysed the sediment variables that could potentially impact taxonomic density in this region, and explored the impacts of these variables on Hill–Simpson diversity and Pielou evenness. In addition, two local datasets were analysed to determine how the diversity indices respond to sediment variables at the local scale. Finally, we outline a strategy for the ecological assessment of sediment quality using diversity indices in local marine areas.

## Results

### Potential responses of diversity indices

Polychaeta was the most diverse class of benthic invertebrates in the regional dataset, and Malacostraca, Bivalvia, and Gastropoda were the next most diverse classes overall (Supplementary Table S1). Benthic invertebrate assemblages were relatively more diverse in the low-frequency group than in the other groups in the regional dataset (see “Materials and methods” for the definition of groups), whereas Polychaeta was dominant in the high-frequency group. The average number of families observed at each location in the regional dataset was 12.4 ± 8.2 (mean ± standard deviation) with a range of 0–36 (Table 1). We set up the low-, intermediate-, and high-frequency groups to have a similar number of families among groups.

**Table 1:** Summary of the regional dataset and results of average generalised linear mixed models (GLMMs). Shown are the effect sizes of log-transformed water content (WC), the standard deviation of random effects (σ), and their ratio, for GLMMs in the overall dataset, and in the low-, intermediate-, and high-frequency groups. σ was significant in the overall dataset and for all frequency groups. WC was significant in the overall dataset and for the low-frequency group, but was not significant in the other two groups.

	No. of families in observation			Effect size in GLMMs		
				WC	σ	WC/σ
	Mean ± S.D	Min	Max	Mean (95% CI)	Mean (95% CI)	
Overall	12.4 ± 8.2	0	36	− 0.34 (− 0.58 to − 0.10)	0.62 (0.46 to 0.78)	− 0.55
Low	3.8 ± 3.9	0	16	− 0.54 (− 0.92 to − 0.16)	0.67 (0.45 to 0.88)	− 0.81
Intermediate	3.9 ± 3.2	0	15	− 0.29 (− 0.74 to 0.15)	0.87 (0.61 to 1.14)	− 0.33
High	4.7 ± 2.5	0	9	− 0.23 (− 0.70 to 0.24)	1.18 (0.84 to 1.51)	− 0.20

**Figure 2:**
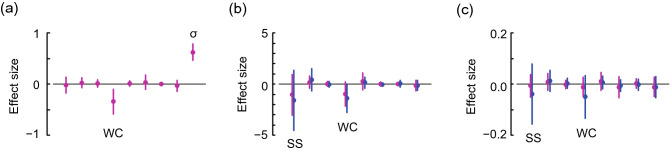
Results of averaged models using the regional dataset. Panels show (**a**) generalised linear mixed model results for the number of families observed, (**b**) generalised linear model results for Hill–Simpson diversity, and (**c**) Pielou evenness. The effect sizes of standardized sample size (SS), latitude, water depth, log-transformed water content (WC), log-transformed median sediment particle size, total organic carbon (TOC), interaction between TOC and the carbon/nitrogen molar ratio, and sediment temperature are shown from left to right in each panel. The standard deviation of estimated random effects (σ) is shown on the right side of (**a**). Circles and bars represent means and 95% confidence intervals, respectively. Magenta and blue indicate results from the overall data (*N* = 65 for the number of families and Hill–Simpson diversity; *N* = 60 for Pielou evenness) and for reliable data from samples with at least 50 individuals (*N* = 36 for both Hill–Simpson diversity and Pielou evenness), respectively.

The sediment characteristic water content (WC, %), ranging from 24.7 to 360%, had significant power to explain the variability of family density in the overall regional dataset (Fig. 2a; the results shown are from an averaged model based on the Akaike information criterion [AIC]; see Supplementary Table S2 for the accepted candidate models). Water depth, latitude, and sample size were not significant. Also, the effect of WC was significantly negative in the low-frequency group (Supplementary Fig. S1). In this group, the ratio of the effect size of WC to the standard deviation of random effects was more than double in absolute value the values in the other groups (Table 1).

Hill–Simpson diversity for the overall data in the regional dataset (*N* = 65) was 4.3 ± 3.1 (mean ± standard deviation). Pielou evenness was 0.71 ± 0.21 for sampling locations where Pielou evenness was defined (*N* = 60). Hill–Simpson diversity and Pielou evenness were 4.8 ± 3.4 and 0.64 ± 0.18 (*N* = 36), respectively, in the reliable data (see Materials and Methods for the definition of reliable and unreliable data), where WC ranged between 32.7 and 263%. In the unreliable data, Hill–Simpson diversity was 3.7 ± 2.5 (*N* = 29) without saturation in the rarefaction curve (Supplementary Fig. S2), and Pielou evenness tended to be greater (0.82 ± 0.20; *N* = 24, where Pielou evenness could be defined) than in the reliable data at the same family density (Supplementary Fig. S2).

The effect size of WC was negative for Hill–Simpson diversity in the analysis of reliable data (Fig. 2b). Among models with low AIC values, the effect size of WC was more negative in models that used only the reliable data than in models that used both reliable and unreliable data (Supplementary Table S3). The confidence interval for Pielou evenness extended farther into the negative range in the analysis of reliable data (Fig. 2c). This extension was produced because low-AIC models including WC selected for reliable data, whereas the explanatory variables varied in analyses with small AICs for the overall data (Supplementary Table S4). Although the effect of WC did not differ significantly from zero, its shift toward the negative means that there was a greater possibility of selecting a model including WC when using the reliable data. Sample coverage was 0.94 ± 0.04 in the set of reliable data (*N* = 36), which was significantly greater than in the unreliable data overall (0.61 ± 0.34; *N* = 29, *t* =  − 5.1, *P* = 2.1 × 10^−5^; Welch two-sample t-test) and in the unreliable data where Pielou evenness was defined (0.74 ± 0.20; *N* = 24, *t* =  − 4.7, *P* = 9.7 × 10^−5^).

### Assessments at two local sites

#### Community structure in Matsunaga Bay

In Matsunaga Bay, the most diverse classes of benthic invertebrates were Polychaeta, Malacostraca, Bivalvia, and Gastropoda (Supplementary Table S1), which was the same as in the regional dataset. The ranges of values of other sediment variables were similar to those in the regional dataset except for sediment temperature (Supplementary Fig. S3). Because this local dataset was acquired in winter (see Appendix S1), sediment temperature (13.9 ± 1.9 °C; mean ± standard deviation) was lower than the temperature in the regional dataset (24.8 ± 3.5 °C; between 10.4 and 30.4 °C).

**Figure 3:**
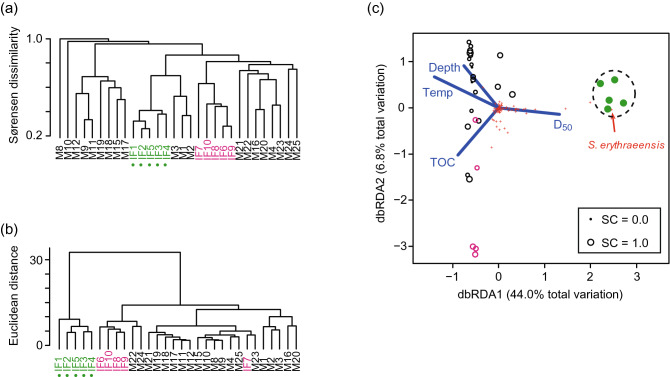
Community structure in Matsunaga Bay. Dendrograms based on (**a**) Sørensen dissimilarity and (**b**) log(*n* + 1) Euclidean distance. (**c**) Model-selection results in the redundancy analysis based on log(*n* + 1) Euclidean distance. Dots in (**a**) and (**b**) indicate reliable observations (data from at least 50 individuals). Green and magenta circles indicate observations from intertidal flats at the mouth of a small river and in the inner bay, respectively. The size of the circles in (**c**) represents sample coverage (SC). Open and closed circles in the panels indicate unreliable (from fewer than 50 individuals) and reliable observations (50 or more individuals), respectively. Red crosses indicate benthic invertebrate species. *Simplisetia erythraeensis* was the most dominant species at the mouth of the small river.

Cluster analysis based on Sørensen dissimilarity did not reveal any apparent differentiation of species compositions in Matsunaga Bay (Fig. 3a); species compositions were similar within the intertidal flat at the mouth of a small river (sample locations IF1–IF5) and within the intertidal flat in the inner bay (IF6–IF10). By contrast, cluster analysis based on Euclidean distance after log(*n* + 1) transformation showed that the community structure at the mouth of the small river was quite different from those at other locations in the bay (Fig. 3b). Distance-based redundancy analysis (dbRDA) showed that the four explanatory variables selected explained 56.9% of total variance in the benthic invertebrate community. The variability in community structure correlated with median sediment particle size (D_50_; 31.6%, *P* = 0.0001) (Fig. 3c), which appears as a difference in structure between the community at the mouth of the small river and the other observations along the first dbRDA coordinate axis. The polychaete *Simplisetia erythraeensis* was the most dominant in the community at the mouth of the small river. The variation in community structure was correlated with temperature (13.0%, *P* = 0.0004), depth (8.0%, *P* = 0.0044), and TOC (4.4%, *P* = 0.042).

Taken together, the cluster analysis and dbRDA for Matsunaga Bay indicate that there is a specific community at the mouth of the small river (hereafter, the “river-mouth community”) with more individuals than at the other locations. The data were reliable for this river-mouth community, but were unreliable at the other 25 locations.

#### Community structure in Nagoya Port

The sediment variables in Nagoya Port had ranges similar to those in the regional dataset (Supplementary Fig. S3). The range of sediment temperatures was within that of the regional dataset because both datasets were obtained in summer (Supplementary Appendix S1).

**Figure 4:**
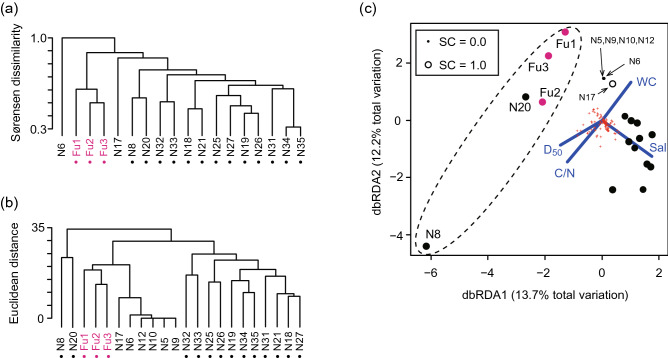
Community structure in Nagoya Port. Panels are the same as in Fig. 3. See Fig. 3 for the meanings of dots and circles in each panel. Magenta circles indicate observations from the Fujimae intertidal flat.

Cluster analysis based on Sørensen dissimilarity was performed for all data from Nagoya Port except for locations N5, N9, N10, and N12, where there were no individuals sampled. The resulting tree showed that at the higher levels, the Fujimae intertidal flat and locations N6 and N17 could be separated from observations at other locations (Fig. 4a). However, the data for N6 and N17 were classified as unreliable. This means that the Fujimae intertidal flat was a habitat type that differed in species composition from other reliable data. The cluster analysis based on log-transformed abundance showed that N8 and N20 were separate specific habitat types in the community structure (Fig. 4b). The second outcome of this analysis is that the habitat type of the Fujimae intertidal flat can be separated from the other 11 locations with reliable data. The dbRDA coordination resulted in four explanatory variables explaining 38.4% of total variance in the benthic invertebrate community at Nagoya Port. Salinity separated the community structures of the Fujimae intertidal flat, N8, and N20 from other locations (Fig. 4c), however, this explained only 11.1% of the variation (*P* = 0.001). The other three explanatory variables also had relatively low contributions to the variability in community structure: the molar carbon to nitrogen ratio (C/N; 11.2%, *P* = 0.016), WC (9.2%, *P* = 0.0006), and D_50_ (6.8%, *P* = 0.039).

Taken together, these analyses suggest that locations N8 and N20 had specific community structures in terms of abundance, and that the Fujimae intertidal flat represents a distinct cluster in terms of species composition and abundance. These locations can be judged to include minor communities (see Materials and Methods). Conversely, the remaining reliable observations (data from 11 locations) were relatively isolated.

#### Responses of diversity indices at two local sites

Because the analysis of the regional dataset showed that WC was the sediment variable with the most impact, we analysed trends in the diversity indices at the two local sites with WC as an explanatory variable.

**Figure 5:**
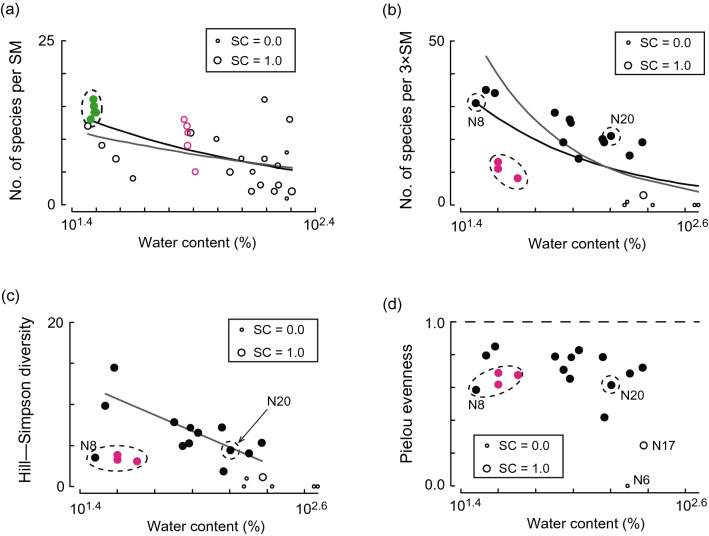
Response of diversity indices at two local sites. Relationships between sediment water content (WC, %) and species density (number of species per Smith–McIntyre bottom sample [SM]) at (**a**) Matsunaga Bay and (**b**) Nagoya Port. The relationships between WC and (**c**) Hill–Simpson diversity and (**d**) Pielou evenness at Nagoya Port. The sizes of circles represent sample coverage (SC). Open and closed circles in the panels indicate unreliable (from fewer than 50 individuals) and reliable (50 or more individuals) observations, respectively. The black line represents the significant regression line for species density for all data in Matsunaga Bay (*t* =  − 5.2, *P* = 2.6 × 10^−7^) and Nagoya Port (*t* =  − 8.8, *P* < 2 × 10^−16^). The grey line in (**a**) and (**b**) represents the significant regression line for species density for all data except those within the dashed line in Matsunaga Bay (*t* =  − 2.7, *P* = 0.0063) and Nagoya Port (*t* =  − 10.8, *P* < 2 × 10^−16^), respectively. The grey line in (**c**) represents the significant regression line for Hill–Simpson diversity for all data except those within the dashed line (*t* =  − 3.7, *P* = 0.0049). In Matsunaga Bay (**a**), green and magenta circles indicate observations from intertidal flats at the mouth of a small river and in the inner bay, respectively. Magenta circles in Nagoya Port (**b**, **c**, and **d**) indicate observations from the Fujimae intertidal flat.

Species density decreased significantly with increasing WC in all data types at the two local sites (Table 2). The slope was similar in the analyses for both the overall data and for the target communities alone in Matsunaga Bay (Fig. 5a). In Nagoya Port, although the response to WC was significant for both data types, the slope was more moderate in species density in the overall data than in the data excluding minor communities (Fig. 5b).

**Table 2:** Summary of generalised linear model results for two local datasets: Matsunaga Bay and Nagoya Port. Shown are the responses of diversity indices to sediment water content. N.S. means that the response was not significant. Results in parentheses are questionable because they are based on unreliable data (fewer than 50 individuals sampled).

Dataset	Data type analysed		Species density		Hill–Simpson diversity		Pielou evenness	
	Data reliability	Community	*N*	Response	*N*	Response	*N*	Response
Matsunaga Bay	Reliable & unreliable data	A river-mouth & target communities	30	Negative	30	(N.S.)	30	(N.S.)
	Unreliable data	Target community	25	Negative	25	(N.S.)	25	(N.S.)
Nagoya Port	Reliable & unreliable data	Specific & target communities	22	Negative	22	(Negative)	18	(N.S.)
	Reliable & unreliable data	Target community	17	Negative	17	(Negative)	13	(Negative)
	Reliable data	Specific & target communities	16	Negative	16	N.S	16	N.S
	Reliable data	Target community	11	Negative	11	Negative	11	N.S

Sample coverage was 0.96 ± 0.02 (mean ± standard deviation) in the river-mouth community (i.e. for reliable data), and 0.61 ± 0.24 at the other 25 locations (i.e. for unreliable data) in Matsunaga Bay. Hill–Simpson diversity was saturated in the river-mouth community but not saturated at the other 25 locations (Supplementary Fig. S4). At the other 25 locations, Pielou evenness tended to be high, approaching 1.0 (Supplementary Fig. S4). In Nagoya Port, Hill–Simpson diversity was almost saturated for the reliable data (Supplementary Fig. S5), where sample coverage was 0.95 ± 0.04. Sample coverage was 0.16 ± 0.46 at the six locations with unreliable data. Pielou evenness was lower at the two locations with unreliable data than at those with reliable data (Supplementary Fig. S5). We analysed the responses of Hill–Simpson diversity and Pielou evenness to WC in Nagoya Port. However, similar analyses were not performed in Matsunaga Bay because of the dearth of reliable data.

The trend of Hill–Simpson diversity with WC was not significant in the analysis of reliable data for both specific and target communities at Nagoya Port, but had a significantly negative slope in the analysis of reliable data for target communities only (Table 2), which excluded the six locations with unreliable data that had a low index at high WC (Fig. 5c). Analyses including both reliable and unreliable data also showed significant negative responses to WC (Table 2). Pielou evenness showed no significant response in the sets of reliable data. However, in the analysis including unreliable data with target communities N6 and N17, which plotted at low Pielou evenness and high WC (Fig. 5d), Pielou evenness showed a negative response to WC (*t* =  − 2.3, *P* = 0.044) (Table 2).

## Discussion

Our analyses of marine invertebrate communities at a regional scale and at two local sites revealed that taxonomic density (i.e. species density) was a sensitive index of marine sediment quality. However, although Hill–Simpson diversity and Pielou evenness were shown to respond to sediment variability in the regional dataset, they could be insensitive or respond falsely when a low number of individuals was observed, and when more than one community co-existed at a local site. These results from two local sites should serve as a point of caution when using diversity indices. Although these indices can provide a good understanding of how communities respond to sediment degradation, it is important to understand how these indices collapse when there is a small number of individuals observed or when the data span multiple co-existing communities. This emphasises the need for better strategies for the ecological assessment of sediment quality based on diversity indices at the local scale in marine areas.

The analyses of the regional dataset show that WC had a larger impact than other variables on the taxonomic density of benthic invertebrate communities, although grain size and organic matter content are also thought to affect benthic invertebrate richness^e.g.^^17,18^. The high contribution of WC likely reflects its physical effects on sediment structure. The optimal range of WC for the burrowing activity of benthic invertebrates is between around 25% WC at the densest (i.e. hardest) and around 40% WC at the loosest (i.e. softest) packing of sediment^19,20^. A WC value exceeding the upper limit of this optimal range could indicate sediment that is too soft for the burrowing activity of benthic invertebrates; this may explain the negative effect of WC on taxonomic density observed in this study.

The relatively high standard deviation of random effects as compared against the effect size of WC in GLMMs suggests that unmeasured variables had a strong effect on taxonomic density. Salinity^21^ and anthropogenic impacts, such as dredging and trawling^13^, are well-known factors that could affect the diversity of benthic invertebrates. However, they were not considered in the regional dataset, because these factors are site- and sampling-location specific, and therefore it was impossible to identify which factors needed to be measured prior to investigation. Our study highlights one advantage of GLMMs, which is the ability to show the effects of these unmeasured factors. The effect size of WC was almost as large as that of the random effects in the low-frequency group (Table 1), which suggests that rare invertebrates were more sensitive to sediment degradation in this group, and that this sensitivity contributed to the overall response of taxonomic density.

The analyses of the regional dataset also showed that an increase in WC caused a decrease in Hill–Simpson diversity and Pielou evenness in the reliable data. This result is consistent with previously identified responses to sediment degradation^6^. Because the low values of these indices occurred in communities with a few dominant species, this suggests that the benthic community was dominated by a few species in soft sediments (i.e. where WC was high).

Similarly, increasing WC was associated with a significant decline in species density at the two local sites (Table 2), and the trend was significant for both reliable and unreliable data. This suggests that WC can be an indicator of benthic invertebrate species density at the local scale. However, it is likely that the trend in species density was not only caused by the effect of sediment softness (as is suggested by our analysis of the regional dataset) but also by other factors. One such factor is anoxia, which has been observed from August to October in the water column above the sediment in Nagoya Port at locations where no individuals were sampled (i.e., N5, N9, N10, and N12)^22^. Similarly, high organic-carbon and trace-metal concentrations have been reported in our study area^23^. These factors could have co-occurred with high WC, and thereby contributed to the decline in species density observed in our study. Because spatial correlations between variables tend to occur at local scales^24^, it is difficult to identify factors that affect species density at this scale. Species density is itself a sensitive indicator; however, if alternatives are needed, parameters that explain variations in species density, such as WC, are recommended for use as a representative variable in local assessment.

WC did not always have a significant negative effect on Hill–Simpson diversity or Pielou evenness at the local scale (Table 2). The significant negative effect of WC on Hill–Simpson diversity identified in the reliable data from Nagoya Port indicates that community structure was dominated by a few species at higher WC, which mirrors the results obtained from the regional dataset. However, WC had no significant effect on Pielou evenness, and even the effect on Hill–Simpson diversity was only significant once locations that have different coexisting community structures (i.e., the Fujimae tidal flat, N8, and N20) were excluded from the analysis. These results mean that these diversity indices are not as sensitive to changes in WC as species density. Conversely, we found questionable significant negative effects of WC on both Hill–Simpson diversity and Pielou evenness when unreliable data were included in the analysis (Table 2). The low values of these indices obtained at high WC likely reflect artefacts in the unreliable data (Fig. 5c, d).

It is important to find and exclude coexisting communities when analysing the effects of sediment degradation on indices of community structure (i.e., Hill–Simpson diversity and Pielou evenness) in a target community. In Matsunaga Bay, the river-mouth community on the intertidal flat was found to have a distinct sediment-particle-size composition compared to other communities in the bay based on multivariate analysis (Fig. 3c). In addition, because the polychaete *Simplisetia erythraeensis* that dominated the river-mouth community can be found in brackish environments (WoRMS: http://www.marinespecies.org/), low salinity (which was not measured in this study) may be a distinguishing feature of this location. Therefore, environmental characteristics such as sediment particle size, salinity, and the location of the intertidal flat likely underlie the spatial variability of community structure in this bay.

Whereas we were able to predict the spatial variability of community structure prior to field sampling in Matsunaga Bay, this was not true in Nagoya Port. Our a priori expectation was that the benthic community on the Fujimae intertidal flat would have a distinct structure because of its location; although this was borne out by the data, we were unable to predict that there would also be distinct community structures at N8 and N20 because of the complex spatial patterning of benthic communities in this area. The explanatory variables we selected (salinity, C/N, WC, and D_50_) explained less than 11% of the total variance in community structure. This weak explanatory power indicates that unmeasured environmental variables may underlie the complex spatial patterning of benthic communities observed in our study, which is typical of the complexity often found in urbanised marine areas^13^.

Although our results demonstrate that excluding distinct coexisting communities from the overall data is important when analysing species density (Fig. 5b) and Hill–Simpson diversity (Fig. 5c), such communities can be difficult to distinguish prior to field sampling. Therefore, *post-hoc* multivariate analysis is needed to distinguish between a target community and other communities. In addition, because diversity indices are affected by both species composition and the proportions of individuals in each taxon, the use of multiple distances between sampling points is recommended to assess how communities differ across space.

**Figure 6:**
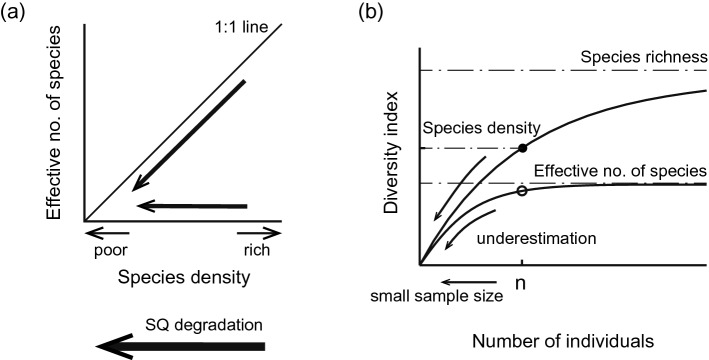
Two mechanisms that can affect the effective number of species (which can be estimated with Hill–Simpson diversity). (**a**) The effective number of species becomes lower at low species density with sufficient sample size. When the degradation of sediment quality (SQ degradation) affects species density but not the density of individuals, the effective number of species decreases as a real response to community structure. However, as shown in (**b**), the effective number of species also becomes lower at small sample size *n*. When SQ degradation affects the density of individuals, the effective number of species might not reflect a real response in community structure.

The unreliability of Hill–Simpson diversity and Pielou evenness values calculated from small sample sizes can be explained by a theoretical framework for the effective number of species^9^. The effective number of species, which reflects the number of dominant species^14^, is predicted to decline or remain unchanged in response to low species density in cases where taxonomic density has a sensitive negative response (Fig. 6a). However, the effective number of species can be underestimated when there is a small sample size (Fig. 6b). This suggests that the questionable negative responses of Hill–Simpson diversity and Pielou evenness (which is calculated from the Shannon index) (Table 2) likely do not reflect real changes in community structure in Nagoya Port, but instead are caused by an artefact that negatively correlates with sediment degradation. However, low Pielou evenness was rarely associated with unreliable data in our study (Appendix S2). Pielou evenness tended to be high, approaching 1.0, in unreliable data from the regional dataset (Supplementary Fig. S2) and Matsunaga Bay (Supplementary Fig. S4). This bias can be explained as a possible result of small sample size. Our results should serve as a warning that false or insensitive responses in Hill–Simpson diversity and Pielou evenness may occur if sample size is insufficient to estimate these indices accurately.

In this study, we used a sample size of 50 individuals as the threshold between reliable and unreliable data. Although a sample-size threshold can be useful when judging whether a sample accurately reflects community structure, the specific value we used was not based on any scientific evidence. In fact, our datasets included several data points classified as “reliable” that were not sufficiently saturated in Hill–Simpson diversity (Supplementary Figs. S2a, S4a, and S5a). Sample coverage is an index that standardises the number of taxa observed by the completeness of the sample^15,25^. The sample coverage of the reliable data was close to 1.0 (complete) and greater than that of the unreliable data in all three datasets used in this study. Although the rarefaction curve is a more direct way to show the estimation accuracy of Hill–Simpson diversity, the simplicity of the sample coverage index (as compared to drawing a rarefaction curve) is an advantage when judging data reliability. In addition, sample coverage is useful when plotting the degree of accuracy in two-dimensional figures, as was done in this study.

When the number of individuals observed, *n*, is not sufficient to estimate the indices of community structure accurately, we can use an extrapolation technique that provides more reliable estimates by doubling the number of individuals observed to 2*n*^9^. Although we did not use this technique in this study because our objective was to explore how small sample sizes affect assessments of marine sediment quality, this technique is a useful solution for practical assessment when the number of individuals observed is not sufficient.

In conclusion, our results show that species density responds sensitively to sediment degradation. By contrast, indices of community structure (i.e. Hill–Simpson diversity and Pielou evenness) were insensitive at the local scale because of masking by multiple coexisting communities, and sometimes produced misleading results because of inaccuracies associated with small sample sizes. Because indices for community structure provide a good understanding of how communities respond to sediment degradation, which cannot be provided by species density, ecological approaches using these indices have merits for assessing sediment quality because they are more realistic under field conditions^3^ and because they reduce uncertainties^26,27^. The potential for misleading and insensitive results must be avoided to keep from diluting these merits. We recommend that these diversity indices for community structure be used in local assessments only if it is possible to obtain a sufficient sample size for accurate estimation, and if co-existing communities can be differentiated before field sampling or by *post-hoc* analysis through sampling at multiple distances.

## Materials and methods

### Sample collection

Surface-sediment samples were obtained separately for use in biological and chemical analysis at each sampling location (Fig. 1). A Smith–McIntyre bottom sampler was used at most locations in all three datasets, but an Ekman–Birge bottom sampler was used at locations where sediment was too soft for a Smith–McIntyre sampler^23^. Sampling depths for these samplers were about 20 cm^28^. The size of the bottom sampler and the number of samplings differed among locations (see Supplementary Tables S5, S6, and S7 for their details).

In sediment samples for biological analysis, benthic invertebrates were obtained by sieving the sample through 1-mm mesh in the field. Invertebrates were preserved in 10% formalin and returned to the laboratory. Biologists in the laboratory identified the taxa sampled and counted the numbers of individuals. Individuals were identified to the lowest taxonomic unit possible and corrected to the accepted names in the World Register of Marine Species (WoRMS: http://www.marinespecies.org/). The differences in the areal size of samples were adjusted for in statistical analyses. However, one data point was excluded from analysis because its sample size was excessively large and biased the analysis too strongly (see Appendix S1).

The sediment samples for chemical analysis were measured for sediment temperature, after which they were immediately chilled with ice and transported to the laboratory. Oxidation–reduction potential (ORP) was also measured in Matsunaga Bay by using an ORP sensor (IM-32P; DKK-TOA Co. Ltd., Tokyo, Japan). Salinity in the water above the sediment surface was measured in Nagoya Port by using multiple-parameter water-quality meters (AAQ; Alec Electronics Co. Ltd., Hyogo, Japan). Chemical analyses of sediments were performed in the laboratory. Water content (WC, %), median sediment particle size (D_50_, mm), total organic carbon (TOC, g kg^−1^ dry wt.), and molar carbon-to-nitrogen ratio (C/N) were measured in all three datasets (see Appendix S3 for details of the chemical analysis). Although WC is measured in units of percent, values can exceed 100% and may reach as high as 1000% in consolidated settled beds^29^.

### Definitions and calculations of diversity indices

Invertebrate specimens were identified to the species level whenever possible for local assessment in Matsunaga Bay and Nagoya Port. However, specimens were only identified to the family level whenever possible in the regional dataset because of possible inconsistencies in identification at the species level (see Appendix S3 for the definitions and calculations of target indices). Hill–Simpson diversity (which takes the number of individuals observed, *n*, as an input variable) was calculated by using the ‘iNEXT’ package^30^ in R^31^. Pielou evenness was calculated by obtaining the Shannon index with the ‘iNEXT’ package and dividing it by log-transformed taxonomic density. We expected only rough estimates of Hill–Simpson diversity and Pielou evenness when the number of individuals observed was small. To assess this potential uncertainty, we distinguished between values calculated by using data on 50 or more individuals (reliable data), and those calculated by using data on fewer than 50 individuals (unreliable data). Finally, sample coverage (a measure of how completely a community has been sampled^15,25^) was also calculated by using the ‘iNEXT’ package to assess whether sample sizes were sufficient.

### Analysis for potential responses of diversity indices

We analysed family density, Hill–Simpson diversity, and Pielou evenness for all data in the regional dataset. In addition, we analysed Hill–Simpson diversity and Pielou evenness calculated from only the reliable data because of the possibility that these indices were less accurate when they included unreliable data. We also analysed the densities for three groups of families categorised as low- (present at ≤ 6 sites out of the total of 65 sites observed), intermediate- (present at between 7 and 25 sites), and high-frequency groups (≥ 26 sites) to find which groups were most affected by sediment quality. These divisions between groups were selected so that the mean numbers of families in observations for each group would be relatively even.

We used a generalised linear model with a random intercept (generalised linear mixed model, GLMM) for these family densities. We applied a Poisson error with a log function for all families and for the low-frequency group. We applied a binomial error with a logistic function for the intermediate- and high-frequency groups. We used a linear model with a Gaussian distribution for the analyses of Hill–Simpson diversity and Pielou evenness. The explanatory variables considered were those related to sediment quality along with several other variables. The sediment-quality variables were WC, D_50_, and TOC, which have been considered in previous studies^16–18^; the interaction between TOC and the C/N ratio, which relates to sediment contaminants^23^; and sediment temperature. Water depth and latitude were also considered because of their known effects on the diversity of marine benthic invertebrates^32–35^. WC and D_50_ were log-transformed before analysis because of their wide ranges.

Areal sampler size was considered as a categorical variable with two possible values: large (i.e. the Smith–McIntyre bottom sampler) or small (the Ekman–Birge bottom sampler). Although overall family density can be adjusted for sampler size by a rarefaction technique, the family densities of the three frequency groups could not be adjusted because the rarefaction technique uses the overall relative abundance of individuals observed. Therefore, to consistently analyse the family density of the overall data and of each frequency group, we treated the areal sampler size as a sampling effect. This effect was included not only for taxonomic density but also for Hill–Simpson diversity and Pielou evenness, to test how their accuracies were affected by sampler size.

We used a technique that averages the candidate models to visualise plausible explanatory variables among many possible good models^36,37^. Before the averaging, we assessed the collinearity and goodness-of-fit of each model (see Appendix S3 for the preparation of candidate models and model averaging). The average estimate of parameters for explanatory variables and their unconditional standard error were calculated by using the Akaike weight^36,37^. Although we show the 95% confidence intervals for the effects of explanatory variables, we discuss them not only in terms of their significance but also in terms of how the possible explanatory variables change between model sets. The maximum likelihoods and standard deviations of regression coefficients were estimated by using the ‘glm’ function for GLMs in R for the linear model, and the R package ‘glmmML’ for the GLMMs^38^.

### Assessment at two local sites

The assessments of two local datasets employed the sediment variable that had the most impact in the regional dataset. Before determining the responses, we tested for differences in community structure in each area. After identifying a community for testing the response, we compared how the response of diversity indices changed for the other communities.

At each local site, we first performed two cluster analyses: Sørensen dissimilarity was used to understand how species compositions differed, and Euclidean distance after a log(*n* + 1) transformation was used to understand how community structures differed in terms of abundance. We applied an unweighted pair-grouping method using arithmetic averages for the clustering based on Sørensen dissimilarity, and Ward’s method for Euclidean distance. Then, we used distance-based redundancy analysis (dbRDA) to understand the relationship between community structure and environmental variables. Water depth and five sediment variables (WC, D_50_, TOC, C/N, and sediment temperature) were used as common explanatory variables for Matsunaga Bay and Nagoya Port. Sample size and ORP were added for Matsunaga Bay. Salinity was added for Nagoya Port. The Euclidean distance after log(*n* + 1) transformation was used to define community patterns. An initial analysis was performed by using forward stepwise tests of all environmental variables to choose the environmental variables that might best explain the patterns^39^. Goodness-of-fit was examined by using the Akaike information criterion (AIC). Models with variance inflation factors of 10 or greater were rejected. We performed dbRDA on the variables selected for the most parsimonious model. The contributions of variables in the selected model were tested by permutational multivariate analysis of variance (PERMANOVA^40^). These analyses revealed the communities that had different species compositions or specific structures in minor environments (i.e. “minor communities”). Cluster analysis was performed by using the ‘hclust’ function in R, and PERMANOVA and dbRDA were performed by using the ‘adonis’ and ‘rda’ functions in the R package ‘vegan’^41^, respectively. PERMANOVA was performed with 9999 permutations.

A community for testing the response to sediment variables was selected as a major community by conducting two cluster analyses and dbRDA in each area. We analysed the trends of species density, Hill–Simpson diversity, and Pielou evenness against the sediment variable that had the most impact in the regional dataset, for the overall data and the reliable data, and for the data excluding minor communities, to test for the effects of unreliable data and minor communities on the diversity indices. We fit a generalised linear model using a Poisson error with a log function for species density, and normal error with a linear function for Hill–Simpson diversity and Pielou evenness. Species density at two sampling locations, M10 and M15, in Matsunaga Bay was adjusted to the number of individuals in an area corresponding to the size of a Smith–McIntyre bottom sampler (see Supplementary Table S6) by using a rarefaction technique^9^. In Nagoya Port, because no individual was obtained at a sampling location, N5, where different samplers were used, no adjustment was necessary.

## Supplementary Information

Supplementary Information 1.

Supplementary Information 2.

## Data Availability

All data generated or analysed during this study are included in this published article and in the Supplementary information.

## References

[1] Magurran AE (2004). Measuring Biological Diversity.

[2] Chariton AA, Pettigrove V, Baird DJ, Simpson SL, Batley GE (2016). Ecological assessment. Sediment Quality Assessment: A Practical Guide.

[3] Chapman PM (2002). Integrating toxicology and ecology: Putting the ‘eco’ into ecotoxicology. Mar. Pollut. Bull..

[4] Wong MC, Dowd M (2015). Patterns in taxonomic and functional diversity of macrobenthic invertebrates across seagrass habitats: A case study in Atlantic Canada. Estuaries Coasts.

[5] Momota K, Hosokawa S (2021). Potential impacts of marine urbanization on benthic macrofaunal diversity. Sci. Rep..

[6] Johnston EL, Roberts DA (2009). Contaminants reduce the richness and evenness of marine communities: A review and meta-analysis. Environ. Pollut..

[7] Gotelli NJ, Colwell RK (2001). Quantifying biodiversity: Procedures and pitfalls in the measurement and comparison of species richness. Ecol. Lett..

[8] Smith W, Grassle F (1977). Sampling properties of a family of diversity measures. Biometrics.

[9] Chao A (2014). Rarefaction and extrapolation with Hill numbers: A framework for sampling and estimation in species diversity studies. Ecol. Monogr..

[10] Gray JS, Elliott M (2009). Ecology of Marine Sediments.

[11] Momota K, Nakaoka M (2017). Influence of different types of sessile epibionts on the community structure of mobile invertebrates in an eelgrass bed. PeerJ.

[12] Momota K, Nakaoka M (2018). Seasonal change in spatial variability of eelgrass epifaunal community in relation to gradients of abiotic and biotic factors. Mar. Ecol..

[13] Todd PA (2019). Towards an urban marine ecology: Characterizing the drivers, patterns and processes of marine ecosystems in coastal cities. Oikos.

[14] Hill MO (1973). Diversity and evenness: A unifying notation and its consequences. Ecology.

[15] Roswell M, Dushoff J, Winfree R (2021). A conceptual guide to measuring species diversity. Oikos.

[16] Hosokawa S (2014). Inference of sediment characteristics relating to the species richness of benthic marine animals, by considering regional and local variabilities. J. Jpn. Soc. Civ. Eng. Ser..

[17] Pearson TH, Rosenberg R (1978). Macrobenthic succession in relation to organic enrichment and pollution of the marine environment. Oceanogr. Mar. Biol. Annu. Rev..

[18] Ellingsen KE (2002). Soft-sediment benthic biodiversity on the continental shelf in relation to environmental variability. Mar. Ecol. Prog. Ser..

[19] Sassa S, Watabe Y (2008). Threshold, optimum and critical geoenvironmental conditions for burrowing activity of sand bubbler crab, *Scopimera globosa*. Mar. Ecol. Prog. Ser..

[20] Sassa S, Watabe Y, Yang S, Kuwae T (2013). Ecological geotechnics: Role of waterfront geoenvironment as habitats in the activities of crabs, bivalves, and birds for biodiversity restoration. Soils Found..

[21] Chapman PM, Wang F (2001). Assessing sediment contamination in estuaries. Environ. Toxicol. Chem..

[22] Sugiyama Y, Yoda M, Harada K (2001). Field survey on anoxic water mass in Nagoya Port. Japanese J. Coast. Enginieering.

[23] Hosokawa S, Naito R, Nakamura Y (2020). Spatial patterns of concentrations of Cu, Zn, Cd, and Pb in marine sediments from Japanese port areas. Reg. Stud. Mar. Sci..

[24] Legendre P, Legendre L (2012). Numerical Ecology. Developments in Environmental Modelling.

[25] Chao A, Jost L (2012). Coverage-based rarefaction and extrapolation: Standardizing samples by completeness rather than size. Ecology.

[26] Batley GE, Burton GA, Chapman PM, Forbes VE (2002). Uncertainties in sediment quality weight-of-evidence (WOE) assessments. Hum. Ecol. Risk Assess..

[27] Batley, G. E. & Simpson, S. L. Introduction. In *Sediment Quality Assessment: A Practical Guide* (eds. Simpson, S. L. & Batley, G. E.) 346 (CSIRO publishing, 2016).

[28] USEPA. *Methods for Collection, Storage and Manipulation of Sediments for Chemical and Toxicological Analyses: Technical manual*. vol. EPA 823-B (2001).

[29] Nakagawa, Y. *Sediment Transport and Near-Bed Dynamics by Currents and Waves in Muddy Environments of Inner Bay: Technical Note of the Port and Airport Research Institute*. vol. 1320 (2016) https://www.pari.go.jp/search-pdf/No1320.pdf (2016).

[30] Hsieh TC, Ma KH, Chao A (2016). iNEXT: An R package for rarefaction and extrapolation of species diversity (Hill numbers). Methods Ecol. Evol..

[31] R Core Team. R: *A Language and Environment for Statistical Computingle* (2020).

[32] Hillebrand H (2004). Strength, slope and variability of marine latitudinal gradients. Mar. Ecol. Prog. Ser..

[33] Gray JS (2002). Species richness of marine soft sediments. Mar. Ecol. Prog. Ser..

[34] Rex MA (1993). Global-scale latitudinal patterns of species diversity in the deep-sea benthos. Nature.

[35] Witman JD, Etter RJ, Smith F (2004). The relationship between regional and local species diversity in marine benthic communities: A global perspective. Proc. Natl. Acad. Sci. U. S. A..

[36] Anderson DR, Burnham KP, Thompson WL (2000). Null hypothesis testing: Problems, prevalence, and an alternative. J. Wildl. Manage..

[37] Burnham KP, Anderson DR (2002). Model Selection and Multimodel Inference: A Practical Information-Theoretic Approach.

[38] Broström G, Holmberg H (2011). Generalized linear models with clustered data: Fixed and random effects models. Comput. Stat. Data Anal..

[39] Oksanen, J. Multivariate analysis of ecological communities in R: Vegan tutorial. https://www.mooreecology.com/uploads/2/4/2/1/24213970/vegantutor.pdf (2015).

[40] Anderson MJ (2001). A new method for non-parametric multivariate analysis of variance. Austral Ecol..

[41] Oksanen, J. *et al.* Community Ecology Package 2.5-7. https://cran.r-project.org/web/packages/vegan/vegan.pdf (2020).

