# Supplementary material for: The use of diversity indices for local assessment of marine sediment quality
Source: Sci Rep. 2021 Jul 22;11:14991. doi: 10.1038/s41598-021-94636-0 (PMC8298509; doi:10.1038/s41598-021-94636-0)
Supplement: Supplementary file 2 — Supplementary Information 2. [file 41598_2021_94636_MOESM2_ESM.docx]

**The use of diversity indices for local assessment of marine sediment quality**

Shinya Hosokawa, Kyosuke Momota, Anthony A. Chariton, Ryoji Naito, Yoshiyuki Nakamura

**Table S1.** Composition of benthic invertebrate communities as determined from three datasets and from the data for the low- (≤6 observations per 65 sites), intermediate- (7–25 observations), and high-frequency groups (≥26 observations) of families in the regional dataset.

**Figure S1.** Results of an averaged model for each frequency group of the regional dataset.

**Figure S2.** Supporting results for the regional analysis.

**Figure S3.** Relationships between explanatory variables in the regional, Matsunaga Bay, and Nagoya Port datasets.

**Figure S4.** Supporting results for the analysis of Matsunaga Bay.

**Figure S5.** Supporting results for the analysis of Nagoya Port.

**Appendix S1: Summary of sampling locations**

**Appendix S2. Sensitivity of Pielou evenness**

**Appendix S3: Detailed explanations for materials and methods**

|  |  | Number of families or species recorded | | | | | |
| --- | --- | --- | --- | --- | --- | --- | --- |
|  |  | Polychaeta | Malacostraca | Bivalvia | Gastropoda | Others | Total |
| Regional | | 38 | 32 | 20 | 14 | 39 | 143 |
|  | Low | 19 | 30 | 15 | 13 | 36 | 113 |
|  | Intermediate | 11 | 2 | 4 | 1 | 3 | 21 |
|  | High | 8 | 0 | 1 | 0 | 0 | 9 |
|  |  |  |  |  |  |  |  |
| Matsunaga Bay | | 39 | 24 | 14 | 14 | 15 | 106 |
|  |  |  |  |  |  |  |  |
| Nagoya Port | | 56 | 15 | 26 | 11 | 14 | 122 |

**Table S1**. Composition of benthic invertebrate communities as determined from three datasets and from the data for the low- (≤6 observations per 65 sites), intermediate- (7–25 observations), and high-frequency groups (≥26 observations) of families in the regional dataset. The values are the numbers of families in the regional dataset and numbers of species in the Matsunaga Bay and Nagoya Port datasets.


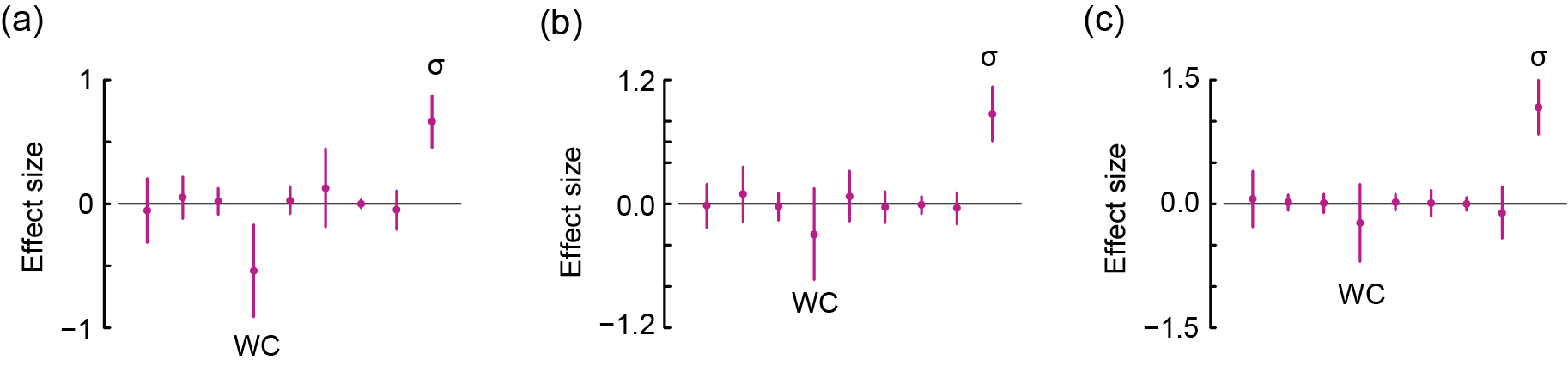


**Figure S1.** Results of an averaged model for each frequency group of the regional dataset. The analyses were performed using the generalized linear mixed model (*N* = 65). Panels show the results for the (a) low-, (b) intermediate-, and (c) high-frequency groups. Shown (left to right in each panel) are the effect sizes of standardized sample size, latitude, water depth, sediment log-transformed water content (WC), log-transformed median sediment particle size, total organic carbon (TOC), interaction between TOC and carbon/nitrogen molar ratio, sediment temperature, and the standard deviation of estimated random effects (σ). Circles and bars represent the means and 95% confidence intervals of effect size, respectively.

**
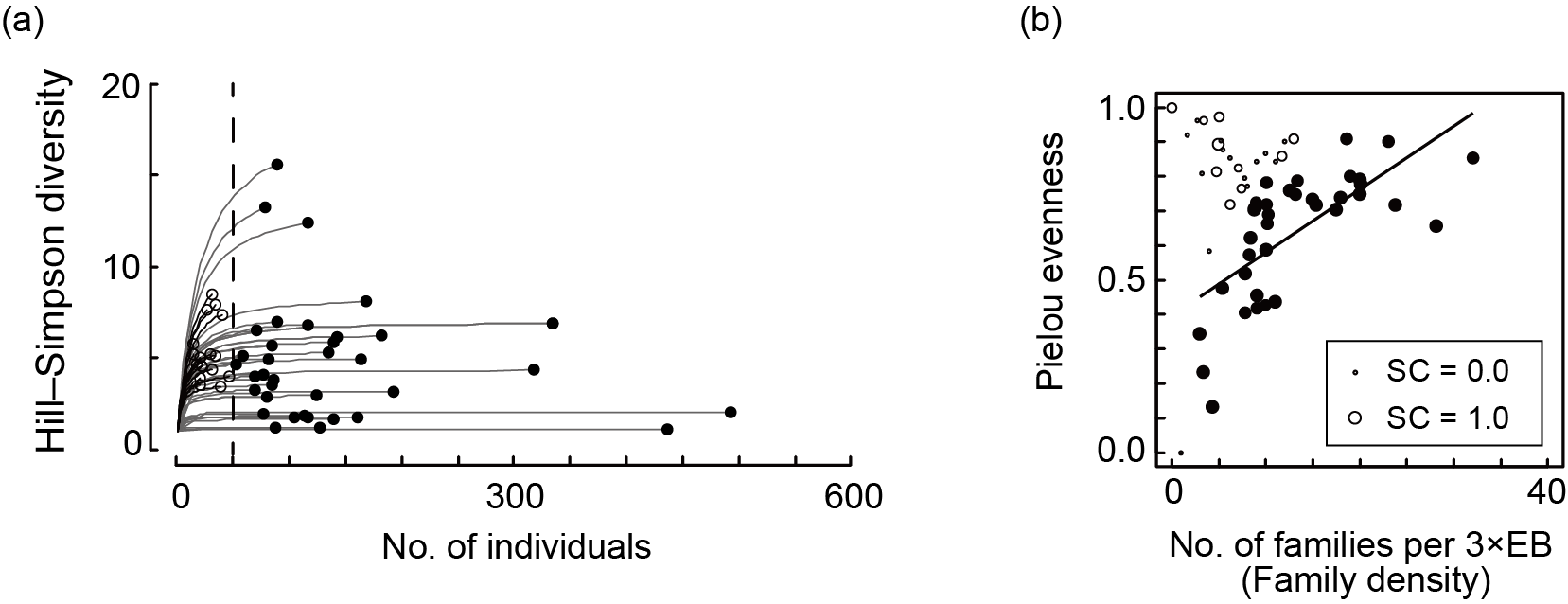
**

**Figure S2.** Supporting results for the regional analysis. (a) Rarefaction curves for Hill–Simpson diversity, and (b) relationship between species density (the number of families per three Ekman–Birge bottom samplers [EB]) and Pielou evenness in the regional dataset. The sizes of circles in (b) reflect sample coverage (SC). Open and closed circles indicate unreliable (from <50 individuals) and reliable (from ≥50 individuals) data, respectively. In (b), the relationship between species density and Pielou evenness was significant for the reliable data (*t* = 5.5, *P* < 0.001).


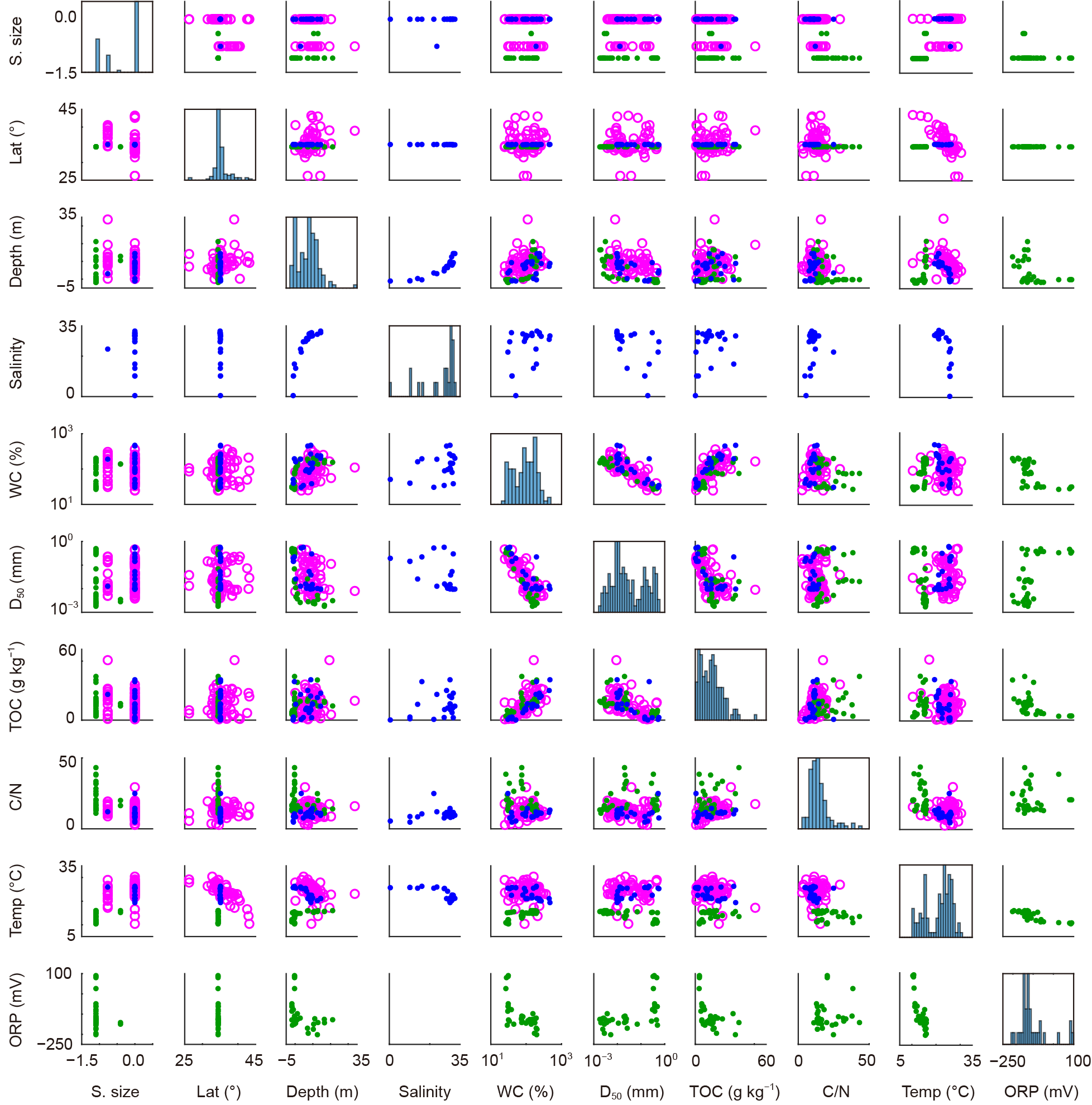


**Figure S3.** Relationships between explanatory variables in the Matsunaga Bay (green) and Nagoya Port (blue) datasets, compared to the regional dataset (magenta). Diagonal bar charts show frequencies for all of the data.


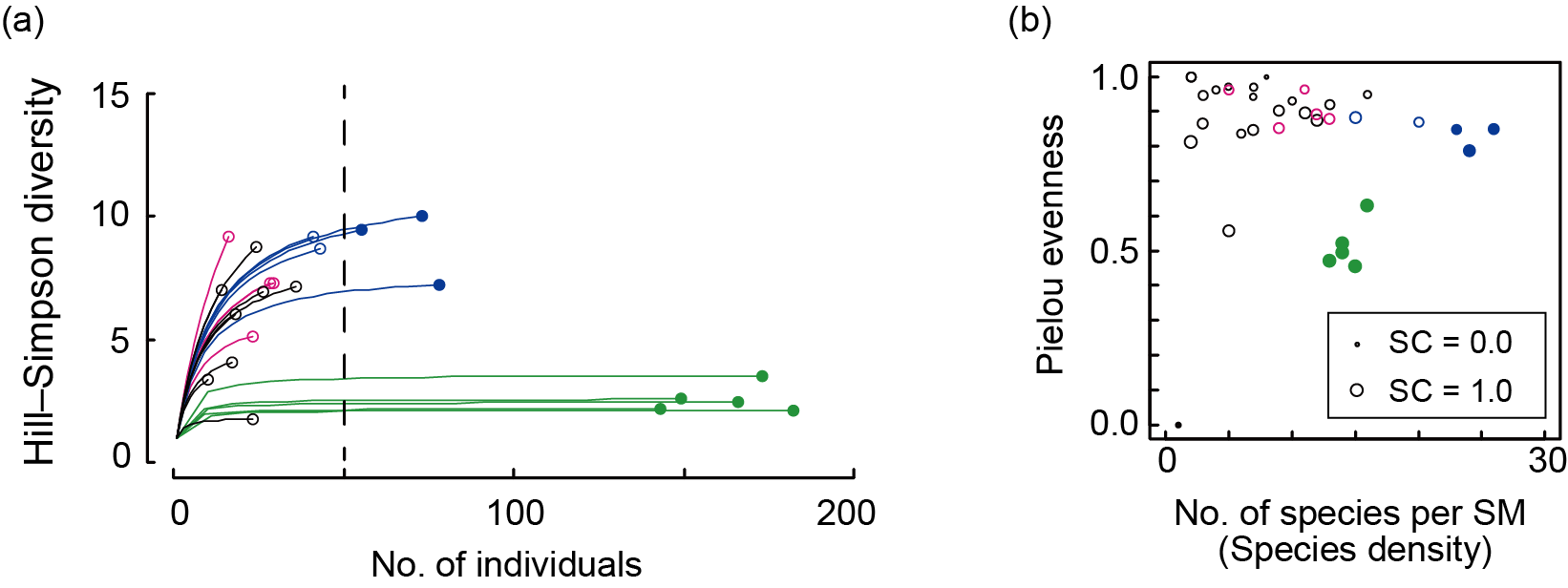


**Figure S4.** Supporting results for the analysis of Matsunaga Bay. (a) Rarefaction curves for Hill–Simpson diversity, and (b) relationship between species density (the number of species per Smith–McIntyre bottom sampler [SM]) and Pielou evenness in the Matsunaga Bay dataset. Green, magenta, and blue represent observations from a tidal flat at the mouth of a small river, one in the inner bay, and from a reference intertidal flat outside the bay, respectively. The sizes of circles in (b) reflect sample coverage (SC). Open and closed circles indicate unreliable (from <50 individuals) and reliable (from ≥50 individuals) data, respectively.


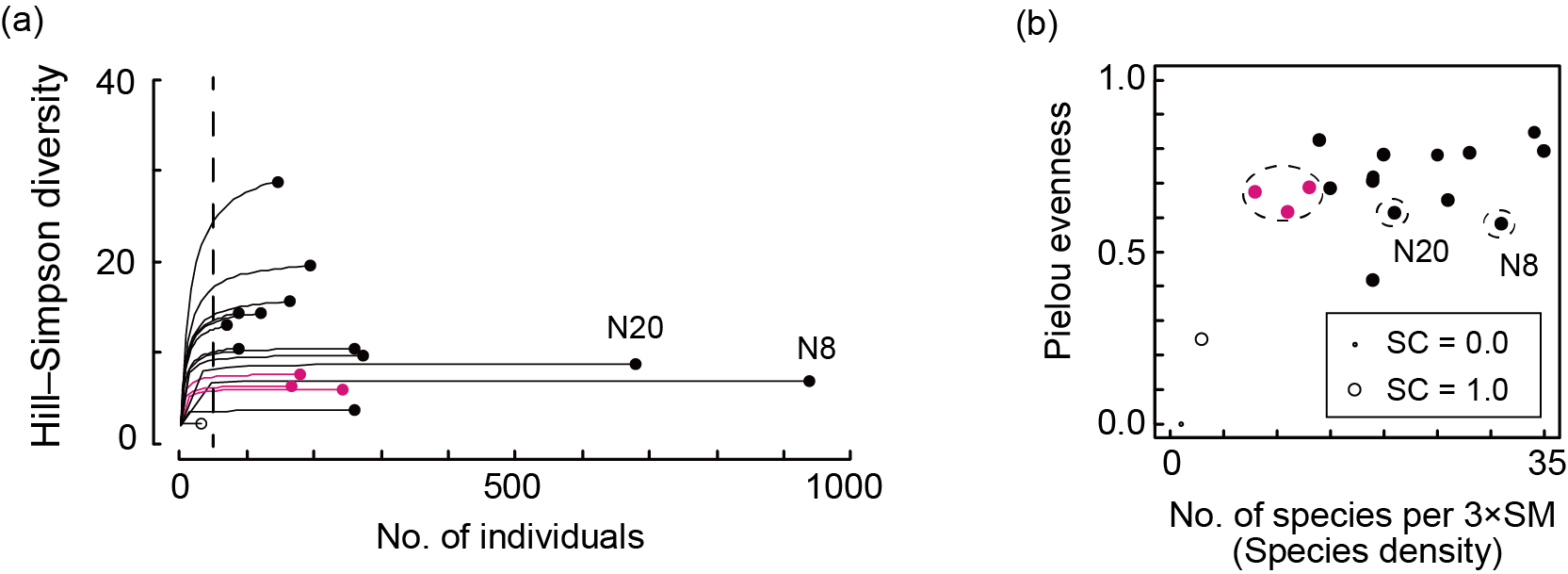


**Figure S5.** Supporting results for the analysis of Nagoya Port. (a) Rarefaction curves for Hill–Simpson diversity, and (b) relationship between species density (the number of species per three Smith–McIntyre bottom samplers [SM]) and Pielou evenness in the Nagoya Port dataset. Magenta data points represent observations at the Fujimae intertidal flat. The sizes of circles in (b) reflect sample coverage (SC). Open and closed circles indicate unreliable and reliable data, respectively.

**Appendix S1: Summary of sampling locations and data treatment**

The regional data were obtained from Japanese port areas by the Ministry of Land, Infrastructure, and Transport (MLIT) between July and September 2002 (**Fig. 1a** in the main text). Because only one or two locations were sampled in each local port, we expected this dataset to be relatively randomized and suitable for finding potential impacts of sediment contamination on benthic invertebrate communities in this region. Two local datasets were selected to include a rural area and an urbanized one. Matsunaga Bay is in a rural area (**Fig. 1b**); it is an enclosed bay in the Seto Inland Sea and includes an intertidal flat at the mouth of a small river (sample locations IF1–IF5 in **Fig. 1b**) and another intertidal flat in the inner bay (locations IF6–IF10). We also analysed data from an intertidal flat outside the bay (locations REF1–REF5), where the benthic community has been well surveyed.

We chose Nagoya Port, the biggest international port in Japan (<https://www.port-of-nagoya.jp/english/aboutport/1001385.html>), as an urbanized area. This area was selected because the Fujimae intertidal flat is located near the port (**Fig. 1c**), and because we expected a complex mosaic of spatial patterns in the sediment habitat, which is typical in urbanized marine areas. The datasets for Matsunaga Bay and Nagoya Port were obtained by the Waterfront Vitalisation and Environment Research Foundation in December 2015, and by a collaboration between the regional MLIT bureau and the Port and Airport Research Institute in July 2008, respectively.

The bottom sampler used and the number of samplings differed among observations within datasets (see **Tables S5**, **S6**, and **S7**). These differences were considered as an explanatory variable in the statistical analyses. However, because the number of sampling sites in Kanmon, in the Japan-wide (regional) dataset, was too large to adjust for sample-size bias in the statistical analyses, these data were excluded from the analyses. In total, we analysed data from 65 sampling locations from the regional dataset, 30 from Matsunaga Bay (with an additional 5 reference samples from outside the bay), and 22 from Nagoya Port.

| **Observation** | **Location** | |  | **Sampling** | | | **Year** | **Month** |
| --- | --- | --- | --- | --- | --- | --- | --- | --- |
|  | **°N** | **°E** |  | **Sampler** | **Sample size (m^2^)** | **No. of biological samplings** |  |  |
| Tomakomai | 42.6003 | 141.7744 |  | SM | 0.0484 | 3 | 2002 | 9 |
| Kushiro | 42.9919 | 144.3183 |  | SM | 0.0484 | 3 | 2002 | 9 |
| Ishikari | 43.2186 | 141.2908 |  | SM | 0.0484 | 3 | 2002 | 9 |
| Sendai | 38.2708 | 141.0134 |  | EB | 0.0225 | 3 | 2002 | 8 |
| Shiogama | 38.3220 | 141.0575 |  | EB | 0.0225 | 3 | 2002 | 8 |
| Hachinohe | 40.5450 | 141.5139 |  | EB | 0.0225 | 3 | 2002 | 8 |
| Ofunato | 39.0550 | 141.7331 |  | EB | 0.0225 | 3 | 2002 | 8 |
| Ishinomaki | 38.3940 | 141.2768 |  | EB | 0.0225 | 3 | 2002 | 8 |
| Akita | 39.7596 | 140.0458 |  | EB | 0.0225 | 3 | 2002 | 8 |
| Noshiro | 40.1989 | 139.9732 |  | EB | 0.0225 | 3 | 2002 | 8 |
| Sakata1 | 38.9289 | 139.8011 |  | EB | 0.0225 | 3 | 2002 | 8 |
| Sakata2 | 38.9433 | 139.7781 |  | EB | 0.0225 | 3 | 2002 | 8 |
| Soma | 37.8437 | 140.9586 |  | EB | 0.0225 | 3 | 2002 | 8 |
| Onahama | 36.9323 | 140.8928 |  | EB | 0.0225 | 3 | 2002 | 8 |
| Nigata-nishi5 | 37.9329 | 139.0610 |  | SM | 0.0484 | 3 | 2002 | 7 |
| Nigata-Higashi | 37.9706 | 139.2234 |  | SM | 0.0484 | 3 | 2002 | 7 |
| Fushikitoyama1 | 36.7738 | 137.1167 |  | SM | 0.0484 | 3 | 2002 | 8 |
| Fushikitoyama2 | 36.7900 | 137.0603 |  | SM | 0.0484 | 3 | 2002 | 8 |
| Fushikitoyama3 | 36.8059 | 137.0654 |  | SM | 0.0484 | 3 | 2002 | 8 |
| Nanao | 37.0597 | 136.9897 |  | SM | 0.0484 | 3 | 2002 | 8 |
| Kanazawa | 36.6274 | 136.6018 |  | SM | 0.0484 | 3 | 2002 | 8 |
| Yokohama | 35.4546 | 139.7090 |  | SM | 0.0484 | 3 | 2002 | 9 |
| Kisarazu | 35.3742 | 139.8883 |  | SM | 0.0484 | 3 | 2002 | 8 |
| Yokosuka | 35.2231 | 139.7179 |  | SM | 0.0484 | 3 | 2002 | 8 |
| TokyowankouC1 | 35.3466 | 139.7476 |  | SM | 0.0484 | 3 | 2002 | 9 |
| TokyowankouC2 | 35.3414 | 139.7689 |  | SM | 0.0484 | 3 | 2002 | 9 |
| MikawaSakae | 34.8006 | 136.9811 |  | SM | 0.0484 | 3 | 2002 | 8 |
| Shimizu | 35.0369 | 138.4800 |  | SM | 0.0484 | 3 | 2002 | 8 |
| Nagoya1 | 35.0258 | 136.8247 |  | SM | 0.0484 | 3 | 2002 | 8 |
| Nagoya2 | 35.0028 | 136.8281 |  | SM | 0.0484 | 3 | 2002 | 8 |
| Yokkaichi | 34.9956 | 136.6706 |  | SM | 0.0484 | 3 | 2002 | 8 |
| Mikawa | 34.7344 | 137.3022 |  | SM | 0.0484 | 3 | 2002 | 8 |
| Kinuura | 34.8581 | 136.9386 |  | SM | 0.0484 | 3 | 2002 | 8 |
| Nakayama | 34.6308 | 136.9911 |  | SM | 0.0484 | 3 | 2002 | 8 |
| Osaka | 34.6422 | 135.3956 |  | EB | 0.0225 | 3 | 2002 | 8 |
| Sakaisenboku | 34.5333 | 135.3944 |  | EB | 0.0225 | 3 | 2002 | 8 |
| Kobe | 34.6903 | 135.2306 |  | EB | 0.0225 | 3 | 2002 | 8 |
| Wakayamashimozu | 34.2194 | 135.1333 |  | SM | 0.0484 | 3 | 2002 | 8 |
| Amagasakinisinomiya | 34.7000 | 135.4000 |  | EB | 0.0225 | 3 | 2002 | 8 |
| Maizuru | 35.4742 | 135.3286 |  | EB | 0.0225 | 3 | 2002 | 8 |
| Sakai | 35.5458 | 133.2667 |  | SM | 0.0484 | 3 | 2002 | 8 |
| Hiroshima | 34.3247 | 132.4044 |  | SM | 0.0484 | 3 | 2002 | 8 |
| Onomichi | 34.4194 | 133.2508 |  | SM | 0.0484 | 3 | 2002 | 8 |
| Kure | 34.2161 | 132.5981 |  | SM | 0.0484 | 3 | 2002 | 8 |
| Fukuyama | 34.4319 | 133.4444 |  | SM | 0.0484 | 3 | 2002 | 8 |
| Tokuyamakudamatsu | 34.0556 | 131.7333 |  | SM | 0.0484 | 3 | 2002 | 8 |
| Iwakuni | 34.1778 | 132.2458 |  | SM | 0.0484 | 3 | 2002 | 8 |
| Ube | 33.9167 | 131.2208 |  | SM | 0.0484 | 3 | 2002 | 8 |
| Tokushimakomatsujima | 33.9936 | 134.6167 |  | SM | 0.0484 | 3 | 2002 | 9 |
| Matsuyama | 33.8481 | 132.6953 |  | SM | 0.0484 | 3 | 2002 | 9 |
| Suzaki | 33.4011 | 133.2922 |  | SM | 0.0484 | 3 | 2002 | 9 |
| Bisanseto | 34.3719 | 133.8125 |  | SM | 0.0484 | 3 | 2002 | 9 |
| Tsuda | 34.2981 | 134.2647 |  | SM | 0.0484 | 3 | 2002 | 9 |
| Kitakyushu1 | 33.9397 | 130.7656 |  | SM | 0.0484 | 3 | 2002 | 8 |
| Kitakyushu2 | 33.9094 | 130.8164 |  | SM | 0.0484 | 3 | 2002 | 8 |
| Kanda | 33.7542 | 131.0408 |  | SM | 0.0484 | 3 | 2002 | 8 |
| Sasebo | 33.1469 | 129.7211 |  | SM | 0.0484 | 3 | 2002 | 8 |
| Kumamoto | 32.7594 | 130.5767 |  | SM | 0.0484 | 3 | 2002 | 8 |
| Yatsushiro | 32.5208 | 130.5261 |  | SM | 0.0484 | 3 | 2002 | 8 |
| Nakatsu | 33.6133 | 131.2531 |  | SM | 0.0484 | 3 | 2002 | 8 |
| Saeki1 | 33.0086 | 131.9094 |  | SM | 0.0484 | 3 | 2002 | 8 |
| Saeki2 | 32.9778 | 131.9231 |  | SM | 0.0484 | 3 | 2002 | 8 |
| Shibushi | 31.4542 | 131.0875 |  | SM | 0.0484 | 3 | 2002 | 8 |
| Kanmon | 33.9769 | 131.0294 |  | SM | 0.0484 | 10 | 2002 | 8 |
| Naha1 | 26.2150 | 127.6591 |  | SM | 0.0484 | 3 | 2002 | 9 |
| Naha2 | 26.2254 | 127.6669 |  | SM | 0.0484 | 3 | 2002 | 9 |

**Table S5.** Summary of data in the Japanese regional dataset. EB and SM refer to Ekman–Birge and Smith–McIntyre bottom samplers, respectively.

| Observation | Location | |  | Sampling | | | Year | Month |
| --- | --- | --- | --- | --- | --- | --- | --- | --- |
|  | **°**N | **°**E |  | Sampler | Sampler size (m^2^) | No. of biological samplings |  |  |
| IF1 | 34.4377 | 133.2424 |  | SM | 0.0484 | 1 | 2015 | 12 |
| IF2 | 34.4378 | 133.2424 |  | SM | 0.0484 | 1 | 2015 | 12 |
| IF3 | 34.4379 | 133.2424 |  | SM | 0.0484 | 1 | 2015 | 12 |
| IF4 | 34.4379 | 133.2425 |  | SM | 0.0484 | 1 | 2015 | 12 |
| IF5 | 34.4378 | 133.2425 |  | SM | 0.0484 | 1 | 2015 | 12 |
| IF6 | 34.4288 | 133.2707 |  | SM | 0.0484 | 1 | 2015 | 12 |
| IF7 | 34.4288 | 133.2707 |  | SM | 0.0484 | 1 | 2015 | 12 |
| IF8 | 34.4287 | 133.2707 |  | SM | 0.0484 | 1 | 2015 | 12 |
| IF9 | 34.4285 | 133.2707 |  | SM | 0.0484 | 1 | 2015 | 12 |
| IF10 | 34.4285 | 133.2708 |  | SM | 0.0484 | 1 | 2015 | 12 |
| REF1 | 34.3914 | 133.2720 |  | SM | 0.0484 | 1 | 2015 | 12 |
| REF2 | 34.3915 | 133.2721 |  | SM | 0.0484 | 1 | 2015 | 12 |
| REF3 | 34.3916 | 133.2722 |  | SM | 0.0484 | 1 | 2015 | 12 |
| REF4 | 34.3917 | 133.2723 |  | SM | 0.0484 | 1 | 2015 | 12 |
| REF5 | 34.3918 | 133.2724 |  | SM | 0.0484 | 1 | 2015 | 12 |
| M1 | 34.4352 | 133.2447 |  | SM | 0.0484 | 1 | 2015 | 12 |
| M2 | 34.4325 | 133.2453 |  | SM | 0.0484 | 1 | 2015 | 12 |
| M3 | 34.4301 | 133.2460 |  | SM | 0.0484 | 1 | 2015 | 12 |
| M4 | 34.4281 | 133.2466 |  | SM | 0.0484 | 1 | 2015 | 12 |
| M8 | 34.4215 | 133.2497 |  | SM | 0.0484 | 1 | 2015 | 12 |
| M9 | 34.4198 | 133.2504 |  | SM | 0.0484 | 1 | 2015 | 12 |
| M10 | 34.4179 | 133.2512 |  | SM | 0.0484 | 2 | 2015 | 12 |
| M11 | 34.4160 | 133.2512 |  | SM | 0.0484 | 1 | 2015 | 12 |
| M12 | 34.4136 | 133.2501 |  | SM | 0.0484 | 1 | 2015 | 12 |
| M15 | 34.4079 | 133.2463 |  | SM | 0.0484 | 2 | 2015 | 12 |
| M16 | 34.4058 | 133.2447 |  | SM | 0.0484 | 1 | 2015 | 12 |
| M17 | 34.4039 | 133.2431 |  | SM | 0.0484 | 1 | 2015 | 12 |
| M18 | 34.4020 | 133.2422 |  | SM | 0.0484 | 1 | 2015 | 12 |
| M19 | 34.3996 | 133.2423 |  | SM | 0.0484 | 1 | 2015 | 12 |
| M20 | 34.4185 | 133.2424 |  | SM | 0.0484 | 1 | 2015 | 12 |
| M21 | 34.4176 | 133.2455 |  | SM | 0.0484 | 1 | 2015 | 12 |
| M22 | 34.4166 | 133.2484 |  | SM | 0.0484 | 1 | 2015 | 12 |
| M23 | 34.4145 | 133.2548 |  | SM | 0.0484 | 1 | 2015 | 12 |
| M24 | 34.4136 | 133.2579 |  | SM | 0.0484 | 1 | 2015 | 12 |
| M25 | 34.4128 | 133.2608 |  | SM | 0.0484 | 1 | 2015 | 12 |

**Table S6.** Summary of data in the Matsunaga Bay dataset. SM, Smith–McIntyre bottom sampler.

| Observation | Location | |  | Sampling | | | Year | Month |
| --- | --- | --- | --- | --- | --- | --- | --- | --- |
|  | **°**N | **°**E |  | Sampler | Sampler size (m^2^) | No. of biological samplings |  |  |
| N5 | 35.0933 | 136.8978 |  | EB | 0.0225 | 3 | 2008 | 7 |
| N6 | 35.0846 | 136.8965 |  | SM | 0.0484 | 3 | 2008 | 7 |
| N8 | 35.0683 | 136.8856 |  | SM | 0.0484 | 3 | 2008 | 7 |
| N9 | 35.0850 | 136.8906 |  | SM | 0.0484 | 3 | 2008 | 7 |
| N10 | 35.0911 | 136.8870 |  | SM | 0.0484 | 3 | 2008 | 7 |
| N12 | 35.0772 | 136.8792 |  | SM | 0.0484 | 3 | 2008 | 7 |
| Fu1 | 35.0753 | 136.8458 |  | SM | 0.0484 | 3 | 2008 | 7 |
| Fu2 | 35.0739 | 136.8311 |  | SM | 0.0484 | 3 | 2008 | 7 |
| Fu3 | 35.0688 | 136.8410 |  | SM | 0.0484 | 3 | 2008 | 7 |
| N17 | 35.0553 | 136.8656 |  | SM | 0.0484 | 3 | 2008 | 7 |
| N18 | 35.0433 | 136.8687 |  | SM | 0.0484 | 3 | 2008 | 7 |
| N19 | 35.0572 | 136.8820 |  | SM | 0.0484 | 3 | 2008 | 7 |
| N20 | 35.0428 | 136.8945 |  | SM | 0.0484 | 3 | 2008 | 7 |
| N21 | 35.0281 | 136.8581 |  | SM | 0.0484 | 3 | 2008 | 7 |
| N25 | 35.0108 | 136.7942 |  | SM | 0.0484 | 3 | 2008 | 7 |
| N26 | 35.0075 | 136.8225 |  | SM | 0.0484 | 3 | 2008 | 7 |
| N27 | 35.0171 | 136.8518 |  | SM | 0.0484 | 3 | 2008 | 7 |
| N31 | 34.9864 | 136.8233 |  | SM | 0.0484 | 3 | 2008 | 7 |
| N32 | 34.9708 | 136.8228 |  | SM | 0.0484 | 3 | 2008 | 7 |
| N33 | 34.9442 | 136.7947 |  | SM | 0.0484 | 3 | 2008 | 7 |
| N34 | 34.9885 | 136.8001 |  | SM | 0.0484 | 3 | 2008 | 7 |
| N35 | 34.9717 | 136.7633 |  | SM | 0.0484 | 3 | 2008 | 7 |

**Table S7.** Summary of data in the Nagoya Port dataset. EB and SM refer to Ekman–Birge and Smith–McIntyre bottom samplers, respectively.

**Appendix S2. Sensitivity of Pielou evenness**

The uncertainty of Pielou evenness was analysed by resampling from the reliable data in all datasets combined (data from ≥50 individuals). The numbers of individuals resampled randomly, *m*, were 5, 10, 20, and 50 for all reliable data. For example, 5 individuals resampled have 7 patterns of Pielou evenness, 1.0, 0.97, 0.961, 0.960, 0.86, 0.72, and 0.0. Also, if the reliable data were from more than 100, 200, or 500 individuals, then these numbers of individuals were also resampled. The resampling of *m* individuals and calculation of Pielou evenness, *J*(*m*), was performed for 101 iterations. We then determined the median value of the simulated Pielou evenness.

The observed Pielou evenness, *J*(*n*), was 0.653 ± 0.165 (mean ± standard deviation), ranging between 0.132 and 0.909 for the reliable data (≥50 individuals). The calculated Pielou evenness, *J*(*m*), increased with decreasing number of individuals sampled in most of the reliable data (**Fig. S6**). Seventy-three percent of the reliable data (44/60 data) had Pielou evenness greater than 0.960 with 5 individuals resampled, *J*(*m* = 5). Only three data points had Pielou evenness of 0.0.


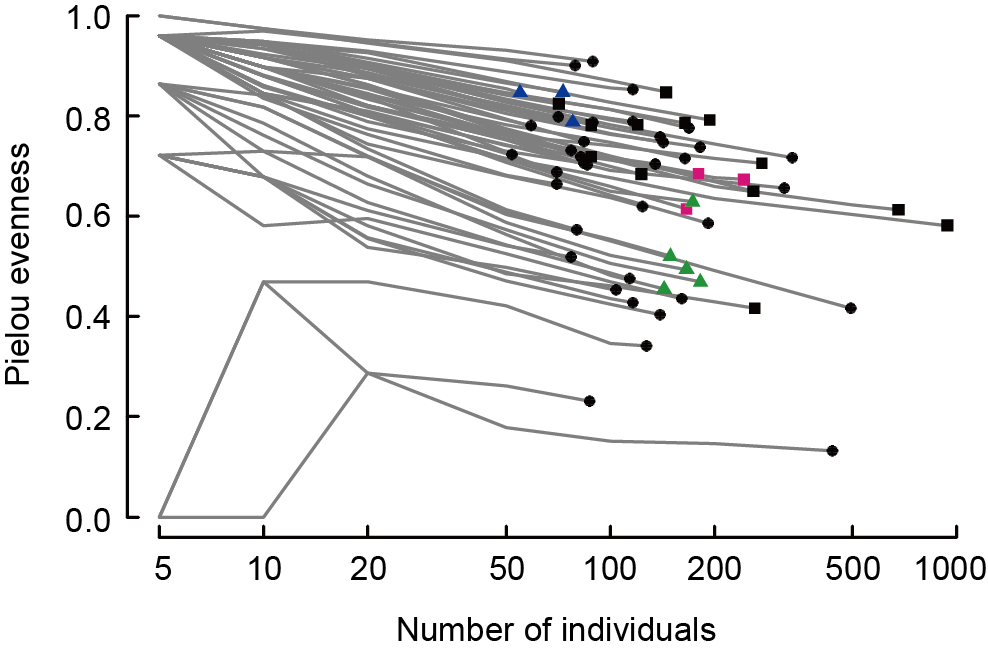


**Figure S6.** Pielou evenness in samples with small numbers of individuals. The figure shows the Pielou evenness, *J*(*m*), for the number of individuals *m* resampled from the observed community. The numbers of individuals resampled were 5, 10, 20, and 50 for all reliable data, and 100, 200, or 500 individuals for reliable data with at least these numbers of individuals. Circles, triangles, and squares represent the observed Pielou evenness in the regional (*N* = 36), Matsunaga Bay (*N* = 8), and Nagoya Port (*N* = 16) datasets, respectively. Coloured symbols represent data from specific intertidal flats: near the mouth of a small river in Matsunaga Bay (green triangles); just outside of Matsunaga Bay (blue triangles); the Fujimae intertidal flat in Nagoya Port (magenta squares) (see **Fig. 1** in main text). Lines show the median values of Pielou evenness from 101 simulations.

**Appendix S3: Detailed explanations for materials and methods**

**Details of chemical analyses.**

Sediment for chemical analysis was obtained separately from the biological samples. The sediment was chilled on ice immediately and transported to the laboratory. All three datasets include sediment temperatures measured immediately after sampling. Oxidation–reduction potential (ORP) was also measured in Matsunaga Bay by using an ORP sensor (IM-32P; DKK-TOA Co. Ltd., Tokyo, Japan). These measurements were performed in subsamples of the sediment. Salinity in the water above the sediment surface was measured in Nagoya Port by using multiple-parameter water-quality meters (AAQ; Alec Electronics Co. Ltd., Hyogo, Japan) during high tide when the intertidal flat was underwater. The locations, water depths, and times of sample acquisition were recorded. The depth to the sediment surface was corrected to an elevation from the chart datum level at the sampling site by using the relationship between the depth at the time of observation and tidal level. Salinity was also measured in Matsunaga Bay (AAQ-RINKO; JFE Advantech Co. Ltd., Hyogo, Japan). Salinities ranged between 30.9 and 32.0 on the practical salinity scale; however, as sampling occurred during ebb tides and did not capture tidal variation, salinity was excluded from all statistical analyses of the Matsunaga Bay data.

Chemical analyses of sediments were performed in the laboratory. Water content (WC, %) and median sediment particle size (D_50_, mm) were measured by using Japanese industrial standard methods. WC is the mass ratio of pore water to sedimentary particles, which ranges between 22.6% in the hardest sediment to 1000% in a consolidating settled bed^1^. Total organic carbon (TOC, g kg^−1^ dry wt.) was measured by using a CHN analyser after acidification of samples. The CHN analyser was also used to measure total organic nitrogen in the regional and Nagoya Port samples and total nitrogen in Matsunaga Bay samples. The molar carbon to nitrogen ratio (C/N) was calculated from TOC and total organic nitrogen in the regional and Nagoya Port samples, and from TOC and total nitrogen in Matsunaga Bay samples.

**Definitions of target indices.**

Taxonomic density, *S_obs_*, is the number of taxa counted within the area of an areal unit sampler. The density is well known to depend on the number of individuals observed, *n*, and shows an increasing curve with increasing number of reference individuals, called a rarefaction curve^2^. The inverse Simpson’s concentration index (Hill–Simpson diversity), *λ*^−1^, and Pielou evenness, *J*, are defined by the true number of taxa, *S*, and the true relative abundance, *p_j_*, of taxon *j* as follows:

$\lambda^{-1}=1/\sum_{j=1}^{S} p_{j}^{2}$ , (1)

$J=H'/\ln S=\left(-\sum_{j=1}^{S} p_{j}\ln p_{j}\right)/\ln S$ . (2)

Here, *H*ʹ is the Shannon index. Hill–Simpson diversity and the exponential of the familiar Shannon index can be integrated with species richness into an equation, which has as a parameter the weighted relative abundance^3^.

The taxonomic levels analysed differed between regional and local datasets. Species were used for local assessment in Matsunaga Bay and Nagoya Port. For the regional dataset, however, the accuracy of identification at the species level might not be consistent because of possible differences in identification among regions. Therefore, family was used as the lowest taxonomic level in the regional dataset. Because the diversity of marine benthic invertebrates was assessed only at the family level^4–6^, we believe that differences in taxonomic classification among locations were not problematic.

**Calculations of the indices.**

To obtain a fair comparison of taxonomic density, it was necessary to consider differences in areal sampler size within the datasets. Taxonomic density in the two local datasets was adjusted to the size of the smaller sampler by a rarefaction technique. However, we did not perform this adjustment in the regional dataset. The justification and detailed methods for how we dealt with the different densities are described in the sections below. Hill–Simpson diversity *λ*(*n*), Shannon index *H*ʹ(*n*), and sample coverage were calculated using the ‘iNEXT’ package^7^ in R. Hill–Simpson diversity was calculated as *λ*(*n*)^−1^. Pielou evenness was calculated by dividing the Shannon index by log-transformed taxonomic density.

Although there is no output from the ‘iNEXT’ function in cases with 0 or 1 individual, we defined Hill–Simpson diversity as 0 for observations with no individual and as 1 for a single individual. Sample coverage was defined as 0 for observations with 0 or 1 individual. Pielou evenness was defined as 0 for observations where a single individual was sampled; however, it was not defined for an observation where no individual was sampled.

**Preparation** **of candidate models and model averaging.**

We constructed candidate models to explore the effects of sediment conditions on diversity indices from combinations of these explanatory variables. However, because collinearity causes unreliable results in linear analysis^8,9^, we rejected as candidate models those with a variance inflation factor of 2 or greater. This strategy for the selection of candidate models was relatively strict than that for the distance-based redundancy analysis at two local sites, because the analysis in the regional dataset purposed to find critical explanatory variables from sediment variables correlating among strongly. In the GLMM, the significance of random effects was assessed by a likelihood ratio test^10^. If the effects were not significant, the candidate model was also rejected if insignificant. A model set was defined as the set of accepted candidate models. The variance inflation factor was calculated as the generalized variance inflation factor (GVIF)^11^ by using the ‘vif’ function in the R package ‘car’. The GVIF for GLMMs was replaced with the GVIF of the generalized linear model that included the same explanatory variables as the GLMM, but without random effects.

The goodness-of-fit of model *i* was assessed by the Akaike information criterion^12^ (AIC). The relative goodness-of-fit of model *i* was assessed as the AIC difference, Δ*_i_* = AIC*_i_* − AIC_min_, where AIC_min_ is the lowest AIC in the set of accepted candidate models. The likelihood of the model given the data was calculated as exp(−Δ*_i_*/2). The Akaike weight of model *i*, *w_i_*, was calculated as the likelihood divided by the sum of the likelihoods for all accepted candidate models.

**References**

1. Nakagawa, Y. *Sediment transport and near-bed dynamics by currents and waves in muddy environments of inner bay*. Technical Note of the Port and Airport Research Institute 1320: https://www.pari.go.jp/search-pdf/No1320.pdf (2016).

2. Smith, W. & Grassle, F. Sampling properties of a family of diversity measures. *Biometrics* **33**, 283–292 (1977).

3. Hill, M. O. Diversity and evenness: a unifying notation and its consequences. *Ecology* **54**, 427–432 (1973).

4. Dethier, M. N. & Schoch, G. C. Taxonomic sufficiency in distinguishing natural spatial patterns on an estuarine shoreline. *Mar. Ecol. Prog. Ser.* **306**, 41–49 (2006).

5. Olsgard, F., Somerfield, P. J. & Carr, M. R. Relationships between taxonomic resolution and data transformations in analyses of a macrobenthic community along an established pollution gradient. *Mar. Ecol. Prog. Ser.* **149**, 173–181 (1997).

6. Somerfield, P. J. & Clarke, K. R. Taxonomic levels, in marine community studies, revisited. *Mar. Ecol. Prog. Ser.* **127**, 113–119 (1995).

7. Hsieh, T. C., Ma, K. H. & Chao, A. iNEXT: an R package for rarefaction and extrapolation of species diversity (Hill numbers). *Methods Ecol. Evol.* **7**, 1451–1456 (2016).

8. Graham, M. H. Confronting multicollinearity in ecological multiple regression. *Ecology* **84**, 2809–2815 (2003).

9. Dormann, C. F. *et al.* Collinearity: A review of methods to deal with it and a simulation study evaluating their performance. *Ecography (Cop.).* **36**, 27–46 (2013).

10. Zuur, A. F., Ieno, E. N. & Smith, G. M. *Analysing Ecological Data*. *Statistics for Biology and Health* (Springer Science, 2007).

11. Fox, J. & Monette, G. Generalized collinearity diagnostics. *J. Am. Stat. Assoc.* **87**, 178–183 (1992).

12. Akaike, H. A new look at the statistical model identification. *IEEE Trans. Automat. Contr.* **AC**-**19**, 716–723 (1974).
